# Sul. Morphia as a Parturient

**Published:** 1871-07

**Authors:** L. D. Robinson


### SUL. MORPHIA AS A PARTURIENT.

Prof. Davis.—*Dear Sir*,—In your April issue, Dr. Vance pretends a reply to my criticism on Dr. Robson's views of morphia as a *parturifacient*, and simply says, *his* experience coincides with the views expressed by Dr. Robson! I admit, that, in natural labor, sul. morphia will act as a parturient—that it will hasten abortion, where it is inevitable; but the question is, *how? What is its modus operandi in such cases?—not, Will it do it?*

Permit me to ask Dr. Vance—1st, What the *modus operandi* of secale cornutum is? 2d, What is the *modus operandi* of sul. morphia upon the parturient patient? These queries I propound, in order to elicit full discussion, and free interchange of views upon this interesting subject, and trust we may *finally* call out the views of the Ed. Examiner on the subject.

L. D. Robinson, M.D.

